# Beyond borders: the choroid plexus-immune communication during neuroinflammation

**DOI:** 10.1038/s41392-024-01997-9

**Published:** 2024-10-08

**Authors:** Anaelle Aurelie Dumas, Adrià Dalmau Gasull, Marco Prinz

**Affiliations:** 1 Institute of Neuropathology, University of Freiburg, Faculty of Medicine, Freiburg, Germany; 2 Signalling Research Centres BIOSS and CIBSS, University of Freiburg, Freiburg, Germany

**Keywords:** Neuroimmunology, Inflammation

In their paper published in Cell,^1^ Xu et al. leveraged single-cell sequencing and cell lineage tracing tools combined with two-photon live imaging to characterise the spatiotemporal immune recruitment and infiltration to the choroid plexus (ChP). They provide seminal insights into the communication between specialised ChP epithelial and macrophage populations, which coordinate the stepwise response to inflammation and its resolution.

**Fig. 1:**
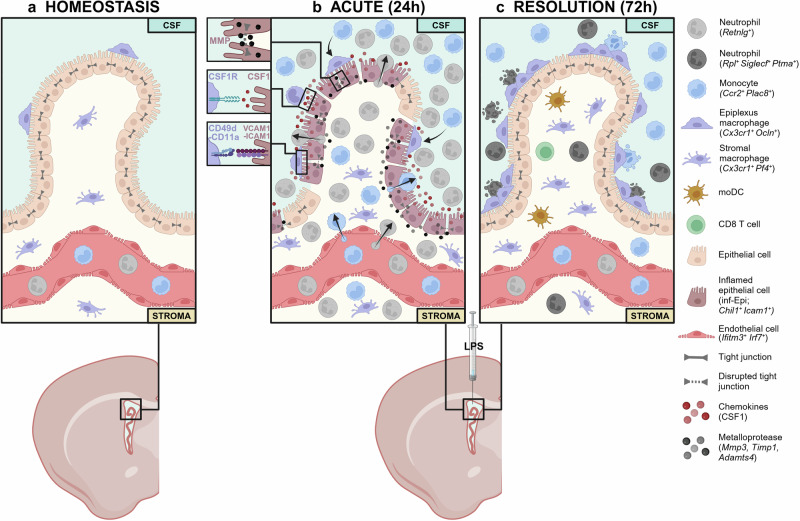
Mechanism of ChP immune response to LPS infection. **a** Immune landscape of ChP under homoeostasis depicting the ChP macrophage populations, which reside in the stromal region and on the apical CSF-containing surface of the epithelial wall. **b** Acute immune response phase to intracerebroventricular delivery of LPS. *Retnlg*^+^ neutrophils and *Ccr2*^*+*^ *Plac8*^*+*^ monocytes enter the ChP via the blood stream into the stroma and through the epithelial barrier into the CSF tissue. *Chil1*^*+*^ *Icam1*^*+*^ inflamed epithelial cells, present during acute inflammation, coordinate immune recruitment and activation in the ChP via the expression of metalloproteases, chemokines and adhesion molecules. **c** By 72 h after LPS treatment, epiplexus macrophages support the resolution of inflammation by phagocytosing immune infiltrates and epithelial cell debris as well as secreting occludin, which contributes to epithelial barrier repair

The blood-brain barrier and cerebrospinal fluid (CSF) serve as sites of immune surveillance contributing to the immune-privileged nature of the central nervous system (CNS). The ChP is a crucial spatial and biological barrier modulating CSF composition and immune entry into the brain. Located in the brain ventricles, the ChP is composed of sheets of epithelial cells surrounding the vascularised stroma where resident immune cells reside.^2^ CNS endogenous macrophages can be found either in the parenchyma as microglia or in CNS interfaces such as leptomeninges, perivascular or ChP. Resident ChP macrophage populations reside in the stromal region and on the apical CSF-containing surface of the epithelial wall (epiplexus macrophages; epiMΦ).^3,4^ Their ontogeny, population maintenance and transcriptomic profile have been partially explored to gain insight into their regulatory role in tissue homoeostasis and disease^3–5^ (Fig. 1a). Under acute inflammation, blood-borne pathogens can gain access to the CNS by disrupting the integrity of its barriers, including that of the ChP.^3,5^ The stepwise immune response taking place in the brain following pathogen entry includes the massive mobilisation of both resident and recruited macrophages across all three compartments: border regions, CSF and parenchyma. Here, Xu et al.^1^ bring clarity to the unique spatiotemporal nature of this response in the ChP in a meningitis-like model.

The authors revealed the ChP-CSF barrier as a site of a prominent immune activation, apparent from tissue swelling and accumulation of leukocytes, which seem to extravasate into ventricles—first in human ChP samples of bacterial meningitis and then in a mouse model of acute brain infection-associated inflammation. This response features neutrophils and monocytes as the first respondents following intracerebroventricular delivery of lipopolysaccharides (LPS) and within 48 h, macrophages became the predominant cell type in both CSF and ChP.

To demonstrate the origin and site of infiltration of leukocytes, the authors used lineage tracing systems combined with two-photon live imaging. Lyz2^+^ S100A9^+^ neutrophils and monocytes showed long-range travel with dynamic translocation from the blood into the ChP stroma as well as from the CSF onto the epiplexus surface. The authors identified the spatial preference of leukocytes to infiltrate at discrete so-called ‘hotspots’ at the ChP-CSF interface where they are able to make their way from the vasculature through the stroma of ChP and the epithelial barriers to enter the CSF. E-selectin expression in postcapillary venules revealed potential sites of entry for leukocyte extravasation into the ChP, as well as breaks in tight junctions between epithelial cells to reach the CSF. For the first time, CX3CR1^+^ macrophages were shown to travel through the CSF and attach to the epithelial outer membrane where they seemed to remain as epiMΦ. They did not display a microglia signature, nor that of border-associated macrophages. Thereby, Xu and colleagues detailed the stepwise ChP immune response to LPS, with neutrophils and monocytes as the first immune cells on site across spatially distinct regions and the rapid expansion of peripheral monocytes alongside CNS macrophages recruited from the blood and CSF (Fig. 1b).

To identify the responsible agent and mechanism by which ChP immune recruitment and infiltration is regulated, the authors subsequently identified temporally enriched populations of stromal and immune cells. They highlight a population of inflammatory epithelial cells (*Chil1*^+^ *Icam1*^+^; inf-Epi) present in early time-points, which are key players in leukocyte recruitment and infiltration. Firstly, inf-Epi upregulate chemokines and matrix remodelling factors. Using a broad matrix metalloproteinase (MMP) inhibitor, the authors demonstrated the role of inf-Epi secreted MMPs in disrupting tight junctions and barrier integrity, facilitating immune infiltration. Secondly, as major producers of colony stimulating factor 1 (CSF1), inf-Epi play a decisive role in the recruitment, differentiation and survival of macrophages in the ChP and CSF environment, validated using CSF1 inhibitor delivered intracerebroventricularely. Lastly, the inf-Epi increased expression of adhesion molecules necessary for the attachment of epiMΦ, demonstrated with the use of antibodies targeted at these adhesion molecules. Together, these findings reveal this newly identified inf-Epi subset, emerging following acute inflammation, as a crucial player in coordinating immune recruitment and activation in the ChP (Fig. 1b).

Having characterised the key players and initial infection response mechanism, the authors focused on the resolution of the inflammation and the repair of the epithelium. Looking at epiMΦ, they could demonstrate their contribution to epithelial healing and restoration to baseline state. Their high motility and phagocytic capacities resulted in the clearing of neutrophils and monocytes, supporting the resolution of inflammation. Moreover, the production of occludin by epiMΦ combined with the cleaning up of epithelial cell debris contributed to epithelial barrier repair. Strikingly, VCAM1/ICAM1 neutralising antibodies, which reduced epiMΦ adhesion to the epithelial membrane, further delayed recovery of occluding by over 5 days (Fig. 1c).

Taken together, this study draws on previous knowledge to reveal the capacity of the ChP to orchestrate an immune response to inflammatory stimuli, followed with tissue repair and resolution of the inflammation, as observed in peripheral tissues. In addition, the special architecture and composition of the ChP endows its unique function as a self-regulating brain barrier. This is evident in the specialised response of a ChP epithelial subset, which utilises CSF-distributed factors to coordinate the recruitment and activation of macrophages across brain barriers and compartments. Although discovered over 100 years ago, the study of epiMΦ remains rudimentary. Xu et al. elegantly demonstrate the significance of their recruitment and adhesion to the CSF-facing surface of the ChP epithelium in the resolution of the inflammatory response as well as in the repair of the ChP barrier. This study leads the field to additional questions on the origin of these CSF macrophages recruited to the ChP, whether they derive from CNS resident populations or differentiate from infiltrating monocytes. Further research will need to explore the exact mode of action, which dictates the attachment of macrophages to the ChP epithelium, and its importance in recovery of tight junctions. The significance of these findings beyond LPS exposure as a model of bacterial infection will benefit from further assessment in a wider range of inflammatory pathogens and in chronic neurological conditions.
